# Novel Mechanism-Based Descriptors for Extreme Ultraviolet-Induced Photoacid Generation: Key Factors Affecting Extreme Ultraviolet Sensitivity

**DOI:** 10.3390/molecules28176244

**Published:** 2023-08-25

**Authors:** Ji Young Park, Hyun-Ji Song, Thanh Cuong Nguyen, Won-Joon Son, Daekeon Kim, Giyoung Song, Suk-Koo Hong, Heeyoung Go, Changmin Park, Inkook Jang, Dae Sin Kim

**Affiliations:** 1 Innovation Center, Samsung Electronics, Samsungjeonja-ro 1, Hwaseong-si 18448, Republic of Korea; 2 Semiconductor R&D Center, Samsung Electronics, Samsungjeonja-ro 1, Hwaseong-si 18448, Republic of Korea

**Keywords:** photoacid generator (PAG), acid-generation mechanism, chemical amplified resist (CAR), triphenylsulfonium (TPS), extreme ultraviolet (EUV) photoresist

## Abstract

Predicting photolithography performance in silico for a given materials combination is essential for developing better patterning processes. However, it is still an extremely daunting task because of the entangled chemistry with multiple reactions among many material components. Herein, we investigated the EUV-induced photochemical reaction mechanism of a model photoacid generator (PAG), triphenylsulfonium cation, using atomiC–Scale materials modeling to elucidate that the acid generation yield strongly depends on two main factors: the lowest unoccupied molecular orbital (LUMO) of PAG cation associated with the electron-trap efficiency ‘before C–S bond dissociation’ and the overall oxidation energy change of rearranged PAG associated with the proton-generation efficiency ‘after C–S bond dissociation’. Furthermore, by considering stepwise reactions accordingly, we developed a two-parameter-based prediction model predicting the exposure dose of the resist, which outperformed the traditional LUMO-based prediction model. Our model suggests that one should not focus only on the LUMO energies but also on the energy change during the rearrangement process of the activated triphenylsulfonium (TPS) species. We also believe that the model is well suited for computational materials screening and/or inverse design of novel PAG materials with high lithographic performances.

## 1. Introduction

In semiconductor device manufacturing, photolithography is a patterning process of a thin film by exposing a photo-sensitive material (photoresist) under a light, then selectively dissolved to fabricate a desired shape on nanometer-length scales. A minimum feature size, which is defined as the minimum size of the elements on a chip, such as the distance between the source and drain on a MOS transistor, could be decreased by decreasing the light’s wavelength used in the lithography system according to the Rayleigh equation [1]. Conventional photolithography that uses deep ultraviolet (DUV) light with the 248 nm wavelength (from the KrF excimer laser) and 193 nm wavelength (from the ArF excimer laser) reduces the minimum feature size down to about 50 nm. Therefore, it is vital to develop a next-generation lithography technology beyond the DUV (KrF/ArF) technology to further scale down the feature size of electronic devices [2]. To that end, the extreme ultraviolet (EUV) photolithography technology, which uses the extreme ultraviolet wavelength of 13.5 nm, has been developed and recently could scale down the feature size to 10 nm in a single exposure patterning.

However, one of the significant challenges in EUV lithography is the ‘stochastic issue’ that the photons are sparsely distributed in the exposed area. Hence, a high EUV exposure dose (low sensitivity) is required to avoid the deterioration of patterning performances, such as the resolution and the critical dimension uniformity (CDU). The following consequences are the lower productivity and higher operation costs of EUV scanners compared to the DUV counterparts [3,4,5]. Therefore, developing high-performance photoresist materials for lower EUV exposure dose (high sensitivity) is critical for high-volume manufacturing. This purpose requires an accurate computational/physical model to predict the EUV exposure dose from the fundamental properties of photoresist materials for high throughput screening, and inverse design would accelerate novel EUV photoresist materials developments.

**Figure 1:**
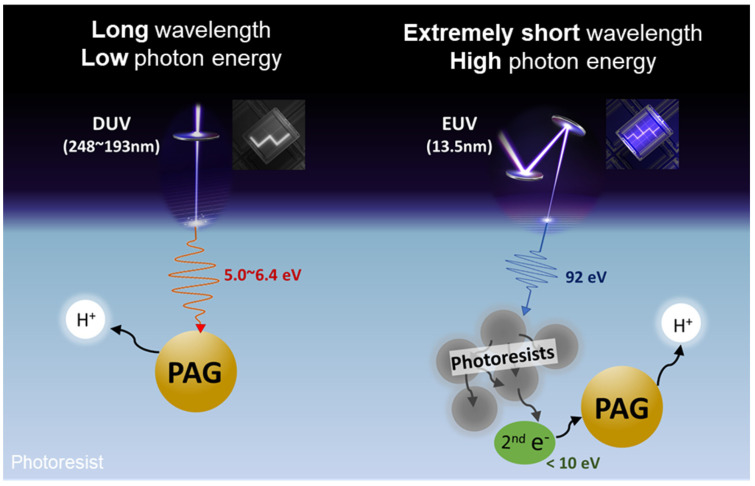
Schematic diagram of DUV and EUV processes.

**Figure 2:**
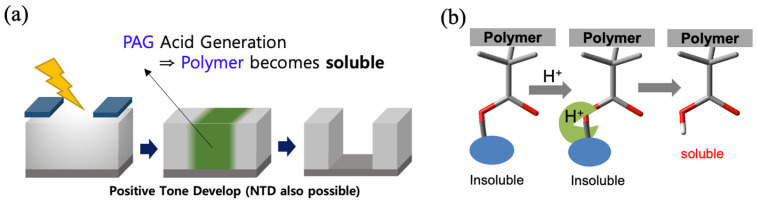
(**a**) Photolithography patterning process and (**b**) solubility change of polymer at the exposed area.

Similar to KrF and ArF photoresists, the typical EUV photoresists are chemically amplified resist (CAR) composites composed of polymers, photoacid generators (PAG), and quenchers. The most commonly utilized PAG is an onium salt based on the triphenylsulfonium cation (Ph_3_S^+^). However, as shown in Figure 1, the high energy of EUV photon (92 eV), compared to that of KrF (5 eV) light and ArF (6.4 eV), results in stark differences in the photochemical processes. Currently, the widely accepted photochemical processes of EUV CAR suggest that the initial incident EUV photons that interact with molecules like as polymer which consist of resist film to generate photoelectrons and subsequent secondary electrons [6,7]. PAG molecules interact with these secondary electrons and finally produce acids. Then, the acid-labile protecting group of the polymer is deprotected to release hydrophilic and acidic functional groups. As a result, the basic aqueous developer has a higher solubility for the exposed and deprotected resist film, as shown in Figure 2. Increasing acid generation yield could afford lower EUV dose without compromising resolution and CDU.

Two reaction pathways for acid generation from sulfonium PAG cations (Ph_3_S^+^-type) are proposed for EUV CAR systems [8,9]. One pathway is the ‘internal excitation’, in which high-energy (10–82 eV) secondary electrons could transfer energy to PAG cations and excite their electrons from the highest-occupied molecular orbitals (HOMO) to the antibonding unoccupied molecular orbitals (LUMO) of the PAG molecules. Then, these excited PAG molecules dissociate to produce acids. The other pathway is ‘electron trapping’, in which the lower-energy (<10 eV) secondary electrons could be trapped by an antibonding lowest-unoccupied molecular orbital (LUMO) of the PAG molecules followed by subsequent PAG decomposition and acid generation, even though recent EUV lithography experiments demonstrated the importance of PAG cation reactions in an acid generation. However, detailed physical and chemical mechanisms of acid generation from PAG cation molecules are still poorly understood.

**Figure 3:**
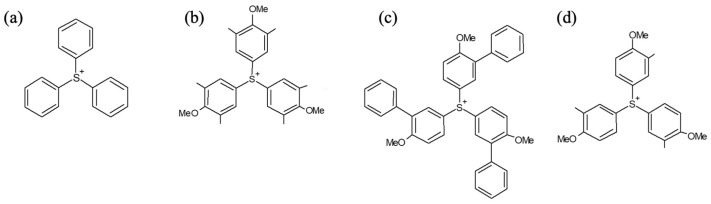
Target PAG cation structures obtained from ref. [10]. (**a**) Triphenylsulfonium (TPS), (**b**) tri(4-methoxy-3,5-dimethylphenyl) sulfonium (MDP), (**c**) tri(4-methoxy-3-methylphenyl) sulfonium(MMP), and (**d**) tri(4-methoxy-3-phenylphenyl)sulfonium) (MPP) with nona-fluorobutanesulfonate as the common conjugate base.

In this work, we aim to develop a EUV sensitivity prediction model by the systematic investigation of the photochemical reactions and acid generation process of PAG cation in both ‘internal excitation’ and ‘electron-trapping’ mechanisms using atomiC–Scale materials modeling. Herein, we targeted triphenylsulfonium (Ph_3_S^+^, TPS) cation, a conventional PAG cation, and its derivatives with symmetrically and unsymmetrically substituted functional groups, as shown in Figure 3. We first tried to reveal the effects of conventional calculated properties (HOMO, LUMO, band gap, pKa) of TPS-based cations on the EUV exposure dose based on the published experimental data (Asakura et al.) [10]. Next, we investigated the entire photo-reaction mechanism of TPS cations under EUV exposure in order to determine the key parameters of the acid generation and EUV exposure dose. We also developed a two-parameter-based prediction model for the PAG performance on EUV exposure dose based on our own experimental data set. Finally, we suggest practical design principles for selecting and synthesizing new EUV PAG materials with superior properties.

## 2. Results and Discussion

### 2.1. Linear Correlation Analysis for DUV(ArF) and EUV Sensitivity

Asakura and coworkers [10] measured three types of the dose-to-size under DUV(ArF) and EUV exposure of four different PAG cations with three-fold symmetry, as shown in Figure 3. Nonafluorobutanesulfonate was the common counter anion of each PAG, and propylene glycol methyl ether acetate (PGMEA) was used as the casting solvent.

**Figure 4:**
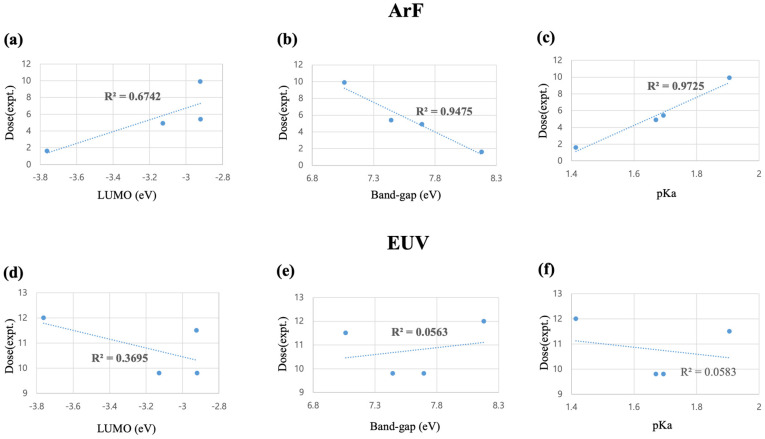
Correlation between the experimental ArF dose from ref. [10] and the DFT-calculated parameters of PAG (**a**) LUMO, (**b**) band gap, and (**c**) pKa (DPS). Correlation between EUV dose with (**d**) LUMO, (**e**) band gap, and (**f**) pKa (DPS) exposure.

Table 1 lists the DFT-calculated properties, including LUMO, band gap energies, and the experimental ArF and EUV exposure dose of PAG cations in Figure 3 from Asakura et al. [10]. In Figure 4a–c, we can observe the linear dependence of experimental ArF exposure dose-to-clear on the LUMO, band gap, and pKa of diphenylsulfide. While ArF dose-to-clear exhibits a moderate dependence on the LUMO of PAG cations with a correlation coefficient of R^2^ = 0.67, band gap and the pKa, the newly suggested parameter by our study, exhibit a higher correlation with the ArF dose-to-clear (R^2^ = 0.95 for the band gap and 0.97 for pKa). On the contrary, there is no correlation between EUV dose-to-size and either band gap (R^2^ = 0.06) or pKa (R^2^ = 0.06). Only the LUMO energy exhibits a low level of correlation with EUV dose with R^2^ = 0.37, as shown in Figure 4d–f. This result demonstrated that the acid generation mechanism in EUV lithography is different from ArF, possibly due to the high energy of EUV photons.

### 2.2. Simulation Study for the Photochemical Reaction Profile of TPS Cation

We mentioned two hypothetical reaction pathways of acid generation from PAG: ‘internal excitation’ and ‘electron trapping’. As the band gap becomes narrower, internal excitation would be easier because electrons occupying HOMO can easily excite into the LUMO level. Furthermore, as the LUMO level becomes lower, a secondary electron would be more easily trapped in the LUMO. Therefore, the efficiency of the ‘internal excitation’ and ‘electron-trapping’ processes should strongly depend on the band gap and LUMO energies. To understand the overall deprotonation process of the PAG cation, we investigated the detailed EUV photochemical reactions of the TPS cation, a conventional PAG cation. For both processes, the TPS cation is postulated to decompose into diphenylsulfide (DPS) and phenyl radical, followed by the formation of rearranged TPS species. Then, the excited or electron-trapped rearranged TPS releases protons to form acids with the counter-anion of the PAG onium salt. Understanding this entire mechanism elucidates the rate-determining step and fundamental properties of acid generation to control the acid generation yield.

**Figure 5:**
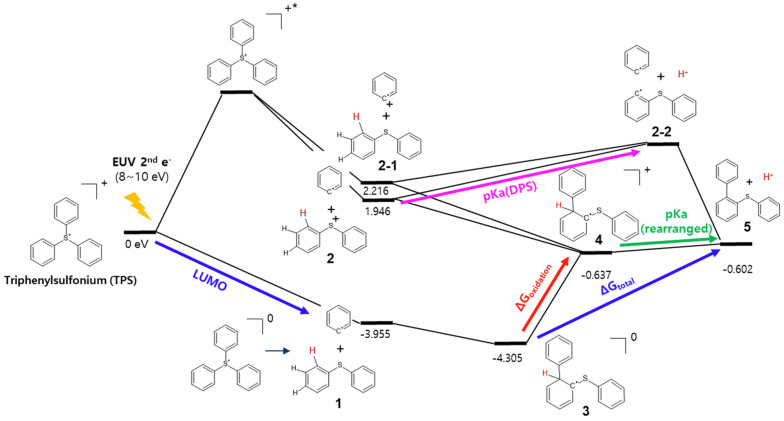
Full reaction mechanism of TPS molecule under EUV. Relative energy values with TPS cation are shown in the eV unit. The * represents the excited state of the molecule.

Figure 5 shows the photochemical reaction energy profile of TPS cation under EUV exposure. In our computational study, ‘electron trapping’ is assumed to occur when the TPS cation captures lower energy secondary electrons (<10 eV). Once the TPS radical is generated, it spontaneously dissociates from the stable DPS molecule and phenyl radical (**1**). On the other hand, ‘internal excitation’ is assumed to occur when the secondary electron transfers energy to an electron by excitation from HOMO to LUMO. This excited TPS cation also undergoes the dissociation into DPS radical cation and a phenyl radical (**2**) or DPS molecule with a phenyl cation (**2-1**). The unstable phenyl radical would soon attack the ortho-position of the phenyl ring in the DPS molecule to form an (ortho)-(phenylthio) biphenyl radical (**3**) [11,12,13]. After or during its generation, the (ortho)-(phenylthio) biphenyl radical (**3**) reduces other PAG cations to form the cation (**4**). The subsequent proton release and recombination between the proton and PAG counter-anion would be the last step of the photo acid generation from the PAG cation (**5**).

Usually, deprotonation energy varies by proton acceptor molecules, but to simplify our model, we introduced another parameter that could represent the proton release ability. Herein, we denote it as pKa(DPS) and pKa(rearranged). We describe the detailed equation in the next section, Section 3—‘Computational Details’. As the acid catalyzes the pattern-forming polarity change reaction of photoresist and the acid generation efficiency is the function of pKa parameters of PAG cation reaction intermediates, it clearly indicates that the pKa parameters can be the quintessential factors to build exposure dose, prediction models.

### 2.3. Two-Parameter Dose-Prediction Model (for TPS Cations from Ref. [10])

As shown in Figure 4, there is a strong correlation between the band gap and ArF dose. It could be translated that the ‘internal excitation’ pathway is dominant under the ArF exposure, and the molecular decomposition of TPS is accelerated by a narrower band gap, which makes electron excitation more accessible from HOMO to LUMO. In the case of the EUV dose, LUMO shows a relatively higher correlation than the band gap and pKa. As we supposed in the previous Section 2.2, secondary electron trapping by TPS cation might proceed under EUV exposure. Unlike the ArF exposure case, only a LUMO level is essential for the ‘electron trapping’ pathway because the low-lying LUMO energy level can be more easily occupied by electrons. However, LUMO is not powerful enough to solely predict EUV dose with a less than moderate correlation of R^2^ = 0.37 (Figure 4d).

To improve the EUV dose predictability, we focused on the post-electron-trap deprotonation processes. The electron charge distribution is significantly affected by the structural changes resulting from decomposition reactions. Therefore, we separated the whole process into ‘before-decomposition’ and ‘after-decomposition.’ in Figure 5. For the ’before-decomposition’ step, the band gap or LUMO level of TPS is evidently decisive, as we already discussed in the Section 2.2. Otherwise, for the ‘after-decomposition’ step, there is an energy barrier from structures **3** to **5**. To represent this energy barrier, we selected four parameters as P_after_: deprotonation efficiency of DPS **2** (pKa(DPS)), deprotonation efficiency of rearranged TPS radical cation **4** (pKa(rearranged)), oxidation potential of rearranged TPS radical **3** (ΔG_oxidation_), and overall energy required to deprotonate the rearranged TPS radical **3** (ΔG_total_). Each energy is obtained as an eV unit by equations proposed in Section 3—‘Computational Details’. We postulated a two-parameter regression model based on combining the parameters obtained from the before- and after-decomposition steps. By the regression analysis with the experimental exposure dose data and the computed molecular properties, we obtain two multiple-linear prediction models:

(1) $$\mathrm{EUV}\mathrm{dose}=\alpha \times \mathrm{LUMO}+\beta \times P_{\mathrm{after}}+\gamma$$

Herein, the above four parameters (pKa(DPS), pKa(rearranged), and ΔG_oxidation_, ΔG_total_) can be a P_after_. We applied this regression model to the test set of four TPS cations in Figure 3. Depending on the selection of P_after_ parameters, a correlation between experimental EUV dose and predicted EUV dose varies, and ΔG_total_ afforded the best correlation (see Table 2).

### 2.4. Extension and Validation of the Two-Parameter Dose-Prediction Model

In the Section 2.2 and Section 2.3, we could conclude that separated reaction steps should be considered: before- and after- C–S bond dissociation. Also, we discovered that four additional parameters, pKa(DPS), pKa(rearranged), ΔG_oxidation_, and ΔG_total_, could be considerable in the ‘after C–S bond dissociation’. When we consider both steps together, the accuracy of EUV dose prediction becomes better for the data sets from ref. [10]. To figure out the most dominant parameter sets, we obtained additional experimental data, the conditions of which are summarized in Table 3. Herein, sets 1, 2, 3, and validation sets contain unsymmetrical TPS molecules. The table shows that only PAG molecules vary in each set while maintaining the same PAG/PDQ/Polymer/solvent type and concentration to verify the correlation between PAG and EUV dose.

**Figure 6:**
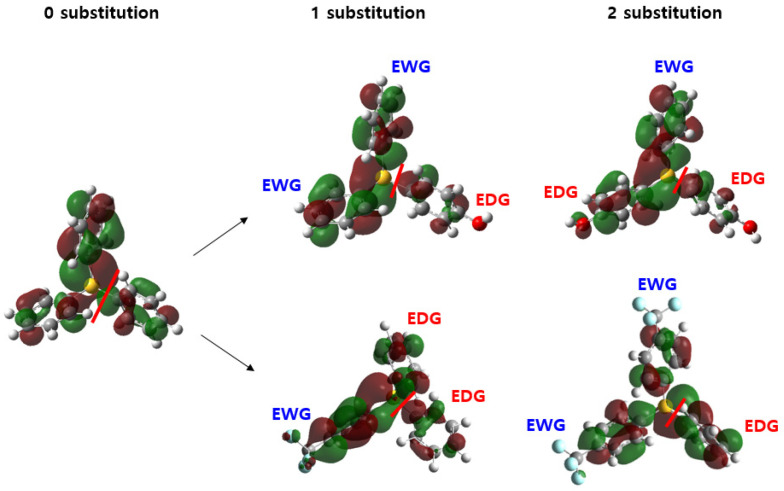
SOMO distribution depends on the number of substituent groups on TPS. The red line shows the dissociation site.

To apply our study to extended sets of PAG cations, including asymmetrically substituted TPS, we need to determine the most likely dissociable phenyl ring of the TPS cation scaffold and the regioselectivity of new C-C bond formation. First, we compared singly occupied molecular orbital (SOMO) or mono- and di-substituted TPS neutral radicals to determine the dissociative pathway of the electron trapping mechanism (Figure 6). The hydroxyl group (-OH) represents the electro-donating substituents, and the trifluoromethane(-CF_3_) represents the electron-withdrawing substituents. While the electron-donating/-withdrawing nature of the substituent influences the conformation of the antibonding orbital responsible for the C–S bond dissociation, the antibonding orbital node locates on the C–S bond of the most electron-rich phenyl ring for all cases of Figure 6. As a result, the relatively more electron-donating group-attached phenyl ring would dissociate from the sulfur center. Because of the conjugation, the C–S bond dissociation would prefer the formation of the substituted phenyl radical and neutral diphenyl sulfide, as shown in structure 1 of Figure 5. The C-C bond formation will follow as the substituted-phenyl radical attacks the more electron-rich site between two phenyl rings of the diphenylsufide.

Table 4 lists the coefficients of determination (R^2^) of two types of analyses: the LUMO-based single-parameter correlation with EUV dose prediction and LUMO- and ΔG_total_-based on a two-parameter EUV dose-prediction model for five sets of photoacid generators (PAG). Based on the above rearrangement scheme, we calculated each PAG molecule’s LUMO, pKa(DPS), pKa(rearranged), ΔG_oxidation_, and ΔG_total_. We performed a two-parameter linear regression model like Equation (1). Results from all models are summarized in the Supporting Information of this paper. We checked the correlation between parameters to avoid over-correlations in the regression model.

Among the four combinations of two-parameter regression models, the pair of LUMO and ΔG_total_ predicted the EUV dose most precisely while maintaining the orthogonality with a low correlation between the two parameters. (See Supporting Information Tables S1–S4). Table 4 compares the LUMO-based dose prediction with the LUMO and ΔG_total_-based dose prediction for three sets. As shown in Table 4, the experimental and predicted dose value correlation is less than 0.7 from the prediction only based on the LUMO. Otherwise, the prediction accuracy increases when we use ΔG_total_ together (R^2^ > 0.7). It is reasonable because deprotonation from the rearranged TPS (structure 3, Figure 5) was determined as the rate-determining step for the electron-trapping hypothesis. During the electron-trapping process, the phenyl ring with electron-donating substituents would be distorted and dissociates spontaneously, as discussed in Section 2.4. Therefore, changing its structure and accepting secondary electrons has restrictions if there is a structural constraint on the TPS. Also, the chemical property of rearranged TPS differs from the original structure, and deprotonation efficiency relies on which substituent group is attached to each ring.

**Figure 7:**
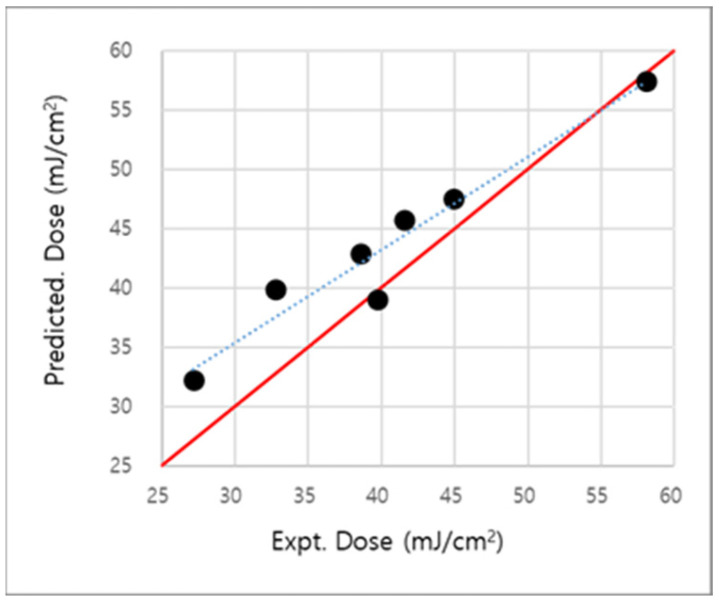
Experimental and predicted EUV dose for seven PAG molecules of the validation set. The dotted line shows a correlation between experiments and prediction. The red line is the baseline for comparing how far from the exact experimental value.

We examined our two-parameter EUV dose-prediction model using seven PAG molecules as the validation set, which has different PAGs; otherwise, the same experimental conditions as set 3. As shown in Figure 7, the predicted EUV dose is well-matched with the experimental EUV dose values with a maximum error of 7.2 mJ/cm^2^. Otherwise, another parameter set predicts dose with R^2^ = 0.08 with pKa(rearranged) and 0.14 with ΔG_oxidation_, respectively. See Table S5 in the Supporting Information to check the details.

(2) $$(\mathrm{Predicted}\mathrm{EUV}\mathrm{dose})=18.56\times \mathrm{LUMO}-194.58\times {\Delta G}_{\mathrm{total}}+717.87$$

### 2.5. Insights on the Design Strategy of the New PAG Molecule

In this section, we want to discuss the novel design strategy of the new PAG molecule. In general, an electron-withdrawing group stabilizes the LUMO level, which might be helpful in lowering the EUV dose. However, at the same time, the electrons-withdrawing group hinders the oxidation, which eventually increases ΔG_total_ and EUV dose. This might provide a compelling rationalization for the negative correlation between LUMO and EUV dose in the data set from ref. [10]. As summarized in Table 4, it follows the following regression model (Equation (3)).

(3) $$\mathrm{EUV}\mathrm{dose}=-2.69\times \mathrm{LUMO}+12.63\times {\Delta G}_{\mathrm{total}}-37.63$$

The five times stronger and positive correlation with ΔG_total_ can be translated that, for this particular resist system, increasing deprotonation efficiency (smaller) would work better than increasing electron-trapping efficiency (lower LUMO). Therefore, we can suggest a stronger electron-donating group-substituted TPS as a better candidate PAG cation for this system.

**Figure 8:**
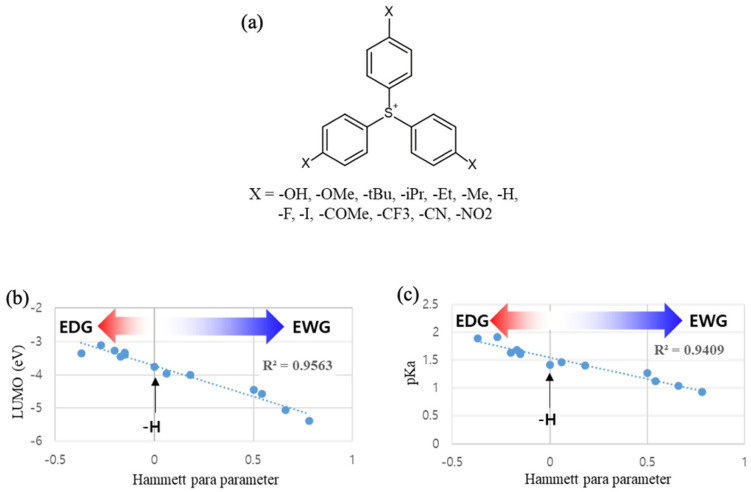
(**a**) List of three fold-symmetrized TPSs with para-substituents. (**b**) Correlation between Hammett para-constant of each substituent (X) and calculated LUMO, and (**c**) correlation between Hammett para-constant and pKa of the rearranged TPS species equivalent to structure 4 in Figure 5.

Additionally, the Hammett constant σ_p_ for para-substituted benzoic acid [14] works as a practical guideline for the TPS substituent effect, exhibiting a strong correlation (R^2^ > 0.95) with both calculated LUMO and pKa values (Figure 8). Considering the trade-off between electron-trapping and deprotonation efficiencies, careful selection of substituent groups for TPS is essential for optimizing photoresist composition.

Herein, we tried to suggest the strategic selection of TPS substituents based on our two-parameter regression model. When the regression model is obtained for a given experimental condition, we can set the direction of the PAG design strategy, either lowering or heightening LUMO or ΔG_total_ by selecting proper phenyl substituents of TPS cations. As shown in Table 4, the coefficient for LUMO (α) and ΔG_total_ (β) varies depending on the experimental conditions and can be classified into three categories.

(1) **α and β >0.** As we discussed previously, satisfying both low LUMO (electron-withdrawing substituents) and low ΔG_total_ (electron-donating substituents) will be frustrated by the trade-off. One might suggest hybrid-type TPS cations with both electron-withdrawing and donating substituents;
(2) **α < 0 and β > 0.** For this photoresist system, electron-donating substituents will be preferred in both LUMO and ΔG_total_-related terms. In this situation, lowering the oxidation potential barrier of rearranged TPS intermediates might be more critical than lowering the LUMO of TPS cations (deprotonation-dominated system);
(3) **α >0 and β < 0.** In this photoresist system, electron-withdrawing substituents could be beneficial to lower the EUV dose by lowering LUMO without significant deterioration of deprotonation efficiency. In this situation, lowering LUMO is more effective than lowering ΔG_total_ (electron-trapping-dominated system).

The factors α and β would vary according to the experimental conditions and concentrations of PAG, PDQ, and polymer. We can also imagine that when the structural distortion of TPS is sterically or electronically forbidden, the TPS cation might have less tendency to accept secondary electrons, and lowering LUMO will not guarantee a lower EUV dose anymore. Also, if a strong basic PDQ is employed, the deprotonation efficiency becomes meaningless, making the electron-trapping step the rate-determining step. The exact contribution of each term should be determined by experiments.

## 3. Computational Details

AtomiC–Scale materials simulations were performed based on the density functional theory (DFT) implemented in the Gaussian16 program package [15]. Gas phase-optimized structures of neutral, cation, and radical molecules/fragments are computed with B3LYP functional [16,17] and cc-pvdz atomic basis set [18]. The Gibbs free energy correction is included by the B3LYP/6-31++G(d) level [19] of single point frequency calculations of gas phase-optimized structures. The solvation effect is approximated by the integral equation formalism variant polarizable continuum model (IEFPCM) [20] with a propylene glycol methyl ether acetate (PGMEA) solvent. The Gibbs free energy and solvation effect calibrated parameters are obtained by following equations.

(4) $$∆G_{\mathrm{solv}}=H-T∆S+\left(∆E_{\mathrm{PGMEA}}-∆E_{\mathrm{gas}}\right)\;=∆G_{\mathrm{gas}}+(∆E_{\mathrm{PGMEA}}-∆E_{\mathrm{gas}})$$

Herein, ΔG_solv_ is the Gibbs free energy, including the solvation effect, ΔG_gas_ is the Gibbs free energy at the gas phase, H is the enthalpy of the gas phase, ΔS is the entropy change of the system, ΔE_PGMEA_ is the internal energy of the PAG molecule surrounded by a PGMEA solvent, and ΔE_gas_ is the internal energy of the PAG molecule in the gas phase. In this paper, all Gibbs free energy terms are corrected values by solvation effect unless otherwise specified.

To obtain deprotonation efficiency and an oxidation barrier for intermediate structures, we defined four equations (Equations (5)–(8)). Each structure numbering is matched with the numbers in Figure 5 in Section 2.1.

(1) pKa of diphenylsulfide (DPS, for structure **2** to **2-2**):
  (5) $$\mathrm{pKa}(\mathrm{DPS})=(∆G_{A^{-}}+∆G_{\mathrm{proton}})-∆G_{HA})$$
(2) pKa of rearranged triphenylsulfonium (TPS, for structures **4** to **5**):
  (6) $$\mathrm{pKa}(\mathrm{rearranged})=(∆G_{\mathrm{struct}5}+∆G_{\mathrm{proton}})-∆G_{\mathrm{struct}4})$$

In both above equations, ∆G_proton_ is calculated from the theoretical Gibbs free energy value (262.4 kcal/mol) of a proton [21], and the single point energy calculation is performed to include the solvation effect in PGMEA(ε = 9.34).

(3) Oxidation potential (for structures **3** to **4**):
  (7) $$∆G_{\mathrm{oxidation}}=∆G_{\mathrm{struct}4}-∆G_{\mathrm{struct}3}$$
(4) Overall energy change (for structures **3** to **5**):
  (8) $$∆G_{\mathrm{total}}=∆G_{\mathrm{oxidation}}+\mathrm{pKa}\left(\mathrm{rearrange}\right)\;=∆G_{\mathrm{struct}4}-∆G_{\mathrm{struct}3}+(∆G_{\mathrm{struct}5}+∆G_{\mathrm{proton}})-∆G_{\mathrm{struct}4})\;=(∆G_{\mathrm{struct}5}-∆G_{\mathrm{struct}3}+∆G_{\mathrm{proton}})$$

## 4. Conclusions

Understanding and analyzing the entangled chemical events and related parameters of EUV-exposed photoresists is mandatory for the theoretical prediction of lithography performances. Herein, we focused on the PAG, an acid-generation component that strongly affects the solubility change of polymer at the EUV exposed area. We figured out that one-parameter, LUMO-based EUV sensitivity prediction has low accuracy. Therefore, we investigated the entire proton generation(deprotonation) and energy profile of the TPS cation, the most commonly employed photo-active molecular scaffold for PAG. We envisioned the reaction into two stages based on the C–S bond dissociation as the critical point, ‘before-decomposition’ and ‘after-decomposition’ of TPS. On the shoulder of the traditional understanding of the ‘before-decomposition’ stage described by LUMO, we introduced four new parameters, pKa(DPS), pKa(rearranged), ΔG_oxidation_, and ΔG_total_, for the ‘after decomposition’ step and developed a two-parameter linear regression model by pairing with LUMO from before the decomposition step. A combination of LUMO and ΔG_total_ gives the best dose-prediction model among the four parameters. Next, we verified our new prediction model with the other experimental data. For three experiments, the experimental dose and predicted dose show a correlation of R^2^= 0.66, 0.67, and 0.91, respectively. Then, we applied this two-parameter model to predict the EUV of new PAG molecules (validation set, Table 4) within less than 7.2 mJ/cm^2^ error range. Finally, we suggested the molecular design strategy based on our two-parameter EUV dose-prediction model.

## Figures and Tables

**Table 1:** Calculated chemical properties of four TPSs (Figure 3) and actual ArF dose-to-clear (E_0_) and EUV dose-to-size (E_size_) (50 nm pitch line-and-space patterning) from Ref. [10].

PAG Cations	HOMO (eV)	LUMO (eV)	Band Gap (eV)	pKa (DPS)	Dose (mJ/cm^2^)	
					ArF (E_0_)	EUV (E_size_)
TPS	−11.942	−3.761	8.181	1.414	1.6	12
MDP	−10.822	−3.127	7.695	1.670	4.9	9.8
MMP	−10.364	−2.920	7.444	1.694	5.4	9.8
MPP	−9.985	−2.922	7.063	1.905	9.9	11.5

**Table 2:** The correlations between experimental and predicted EUV dose, depending on the *P_after_* for Equation (1).

LUMO with	pKa (DPS)	pKa (Rearranged)	ΔG_oxidation_	ΔG_total_
R^2^	0.85	0.95	0.52	0.99

**Table 3:** Conditions of experimental data used in ref. [10] and our study.

Experimental Sets	Ref. [10]	Set 1	Set 2	Set 3	Validation Set
Number of PAG cations	4	9	9	8	7
PAG concentration	4% (polymer:100)	Condition 1	Condition 1	Condition 2	Condition 2
PDQ type	N-1-adamantyl lacetamine	Type1	Type2	Type1	Type1
PDQ concentration	0.27% (polymer:100)	Condition 1	Condition 1	Condition 2	Condition 2
Polymer	PHS/poly(methacrylates) co-polymer	Condition 1	Condition 1	Condition 1	Condition 1
Solvent	PGMEA:Ethyl acetate (7:3)	PGMEA:Ethyl acetate (7:3)	PGMEA:Ethyl acetate (7:3)	PGMEA:Ethyl acetate (7:3)	PGMEA:Ethyl acetate (7:3)

**Table 4:** The single-parameter, LUMO-based correlation with EUV dose and two-parameter, LUMO- and ΔG_total_-based EUV dose-prediction models of photoacid generators (PAG).

LUMO Single-Parameter Correlation with EUV Dose						
Sets of PAG		Ref. [10]	Set 1	Set 2	Set 3	Validation Set
R^2^(LUMO vs experimental EUV dose)		0.49	0.07	0.63	0.83	0.42
LUMO and ΔG_total_ based regression model for EUV dose prediction						
R^2^(experimental vs predicted EUV dose)		0.518	0.66	0.67	0.91	0.94
Maximum difference in dose (experimental—predicted)		11.33	5.70	8.00	5.15	7.15
Correlation between LUMO and ΔG_total_		0.42	0.38	0.55	0.34	0.03
Regression Model Coefficient	α	−2.69	17.86	−16.18	18.56	18.56
	β	12.63	−133.86	−251.49	−194.58	−194.58
	γ	−37.63	528.78	788.71	717.87	717.87

## Data Availability

Restrictions apply to the availability of these data. Data were obtained from the Samsung Advanced Institute of Technology and are available from Suk Koo Hong with the permission of the Samsung Advanced Institute of Technology.

## Supplementary Materials

The following supporting information can be downloaded at: https://www.mdpi.com/article/10.3390/molecules28176244/s1, Table S1. Parameters for regression model and the coefficient of determination between experimental and theoretically predicted EUV dose values for structures in ref. [10]. Correlation between LUMO and pKa(DPS), pKa(arranged), ΔG_oxidation_, and ΔG_total_ are also shown to avoid multiple correlations between parameters. Table S2. Parameters for regression model and the coefficient of determination between experimental and theoretically predicted EUV dose values for structures in set1. Correlation between LUMO and pKa(DPS), pKa(arranged), ΔG_oxidation_, and ΔG_total_ are also shown to avoid multiple correlations between parameters. Table S3. Parameters for regression model and the coefficient of determination between experimental and theoretically predicted EUV dose values for structures in set2. Correlation between LUMO and pKa(DPS), pKa(arranged), ΔG_oxidation_, and ΔG_total_ are also shown to avoid multiple correlations between parameters. Table S4. Parameters for regression model and the coefficient of determination between experimental and theoretically predicted EUV dose values for structures in the set3. Correlation between LUMO and pKa(DPS), pKa(arranged), ΔG_oxidation_, and ΔG_total_ are also shown to avoid multiple correlations between parameters. Table S5. Parameters for regression model and the coefficient of determination between experimental and theoretically predicted EUV dose values for structures in the validation set. Correlation between LUMO and pKa(DPS), pKa(arranged), ΔG_oxidation_, and ΔG_total_ are also shown to avoid multiple correlations between parameters.

## Sample Availability

Samples of the compounds are not available from the authors.
