# Supplementary material for: Novel Mechanism-Based Descriptors for Extreme Ultraviolet-Induced Photoacid Generation: Key Factors Affecting Extreme Ultraviolet Sensitivity
Source: Molecules. 2023 Aug 25;28(17):6244. doi: 10.3390/molecules28176244 (PMC10488646; doi:10.3390/molecules28176244)
Supplement: Supplementary file 1 [file molecules-28-06244-s001.zip › molecules-2560189-supplementary.pdf]

Supporting Information

Correlation between parameters and coefficients for regression model

Each table is coefficients obtained from the two-parameter regression model ($\mathbf{Dose} = \boldsymbol{\alpha} \times \mathbf{LUMO} - \boldsymbol{\beta} \times P_{after} + \boldsymbol{\gamma}$) using data set from reference XX, Set 1 (9 PAG molecules, PDQ-2, Expt. condition 1), Set 2 (9 PAG molecules, PDQ-3, Expt. condition 1), and Set 3 (8 PAG molecules, PDQ-2, Expt. condition 2). Because of the confidential reason, structures for set 1, 2, and 3 are not provided.

Table S1. Parameters for regression model and the coefficient of determination between experimental and theoretically predicted EUV dose value for structures in the reference 10. Correlation between LUMO and pKa(DPS), pKa(arranged), $\Delta G_{oxidation}$, and ΔG_{total} are also shown to avoid multiple correlations between parameters.

LUMO with	pKa(DPS)	pKa(arranged)	Oxidation Pot. $\Delta G_{oxidation}$	Total Oxidation E ΔG_{total}
R^2(Prediction)	0.85	0.95	0.99	0.518
Max diff(Dose, mJ/cm^2)	6.57	3.41	1.03	11.33
Correlation with LUMO	0.81	0.56	0.26	0.42
α	-5.84	0.71	-0.41	-2.69
β	9.01	-13.21	11.35	12.63
γ	-22.87	5.19	-33.02	-37.63

Table S2. Parameters for regression model and the coefficient of determination between experimental and theoretically predicted EUV dose value for structures in set1. Correlation between LUMO and pKa(DPS), pKa(arranged), $\Delta G_{oxidation}$, and ΔG_{total} are also shown to avoid multiple correlations between parameters.

LUMO with	pKa(DPS)	pKa(arranged)	Oxidation Pot. $\Delta G_{oxidation}$	Total Oxidation E ΔG_{total}
R^2(Prediction)	0.70	0.77	0.73	0.66
Max diff(Dose, mJ/cm^2)	9.23	7.03	7.21	-13.115
Correlation with LUMO	0.39	0.63	0.71	0.38
α	27.75	35.61	35.95	17.86
β	-11.59	-87.09	74.99	-133.86
γ	166.77	115.96	-105.95	528.78

Table S3. Parameters for regression model and the coefficient of determination between experimental and theoretically predicted EUV dose value for structures in set2. Correlation between LUMO and pKa(DPS), pKa(arranged), $\Delta G_{oxidation}$, and ΔG_{total} are also shown to avoid multiple correlations between parameters.

LUMO with	pKa(DPS)	pKa(arranged)	Oxidation Pot. $\Delta G_{oxidation}$	Total Oxidation E ΔG_{total}
R^2(Prediction)	0.14	0.07	0.09	0.67
Max diff(Dose, mJ/cm^2)	8.75	13.57	14.88	8.00
Correlation with LUMO	0.28	0.05	0.19	0.55
α	12.21	7.93	5.67	-16.18
β	-33.16	-5.02	-11.78	-251.49
γ	161.07	96.61	137.71	788.71

Table S4. Parameters for regression model and the coefficient of determination between experimental and theoretically predicted EUV dose value for structures in the set3. Correlation between LUMO and pKa(DPS), pKa(arranged), $\Delta G_{oxidation}$, and ΔG_{total} are also shown to avoid multiple correlations between parameters.

LUMO with	pKa(DPS)	pKa(arranged)	Oxidation Pot. $\Delta G_{oxidation}$	Total Oxidation E ΔG_{total}
R^2(Prediction)	0.87	0.87	0.83	0.91
Max diff(Dose, mJ/cm^2)	6.01	4.99	7.07	5.15
Correlation with LUMO	0.73	0.75	0.88	0.34
α	15.16	33.15	29.83	18.56
β	25.04	-70.18	37.36	-194.58
γ	67.27	119.20	15.09	717.87

Table S5. Parameters for regression model and the coefficient of determination between experimental and theoretically predicted EUV dose value for structures in the validation set. Correlation between LUMO and pKa(DPS), pKa(arranged), $\Delta G_{oxidation}$, and ΔG_{total} are also shown to avoid multiple correlations between parameters.

LUMO with	pKa(DPS)	pKa(arranged)	Oxidation Pot. $\Delta G_{oxidation}$	Total Oxidation E ΔG_{total}
R^2(Prediction)		0.08	0.14	0.93
Max diff(Dose, mJ/cm^2)		42.74	37.35	7.15
Correlation with LUMO		0.33	0.35	0.05
α	15.16	33.15	29.83	18.56
β	25.04	-70.18	37.36	-194.58
γ	67.27	119.20	15.09	717.87
